# Retraction: Listerial Meningitis and Brain Abscess With Coexisting COVID-19 Infection in a Young, Immunocompetent Male: A Case Report

**DOI:** 10.7759/cureus.r72

**Published:** 2023-06-05

**Authors:** Syed Faqeer Hussain Bokhari, Huma Sattar, Shaun Abid, Syeda Rabab Jaffer, Samar Sajid

**Affiliations:** 1 Medicine and Surgery, Mayo Hospital, Lahore, PAK; 2 Internal Medicine, Sharif Medical & Dental College, Lahore, PAK; 3 Internal Medicine, Allama Iqbal Medical College, Lahore, PAK; 4 Internal Medicine, Dow University of Health Sciences, Karachi, PAK; 5 Medicine, Dow University of Health Sciences, Karachi, PAK

This article has been retracted due to plagiarism. The journal was contacted by Dr. Vincent Dong with the allegation that this article duplicates much of his prior work found here: https://www.atsjournals.org/doi/pdf/10.1164/ajrccm-conference.2023.207.1_MeetingAbstracts.A5278

The authors were contacted and admitted to the duplication, but maintain it was due to a mistake when handling the lab reports and images. Regardless of intention, Dr. Dong's work has clearly been duplicated and republished and therefore we have made the decision to retract this article. Cureus greatly regrets that the duplication was not detected by our plagiarism checking tool prior to publication.

